# Cardiac magnetic resonance features and outcomes of patients with non-compaction cardiomyopathy – A retrospective follow-up from Pakistan

**DOI:** 10.1016/j.amsu.2022.103962

**Published:** 2022-06-17

**Authors:** Pirbhat Shams, Fateh Ali Tipoo

**Affiliations:** Section of Cardiology, Department of Medicine, The Aga Khan University Hospital, Karachi, Pakistan

**Keywords:** Cardiomyopathy, LV non-compaction, South-Asia, Pakistan, Dilated cardiomyopathy, Non-ischemic cardiomyopathy

## Abstract

**Background:**

The exact prevalence of left ventricle non-compaction cardiomyopathy (LVNC) in south Asians is not known and phenotypic CMR characteristics, clinical features, and outcomes of LVNC remain unknown for the SA population.

**Objective:**

To evaluate clinical characteristics, cardiac magnetic resonance imaging features, and outcomes of patients with left ventricle non-compaction.

**Methods:**

This was a retrospective study of 294 patients undergoing cardiac MRI (CMR) for evaluation of cardiomyopathy from 2011 to 2020. Patients were stratified based on the presence or absence of left ventricle non-compaction (LVNC). Clinical characteristics, CMR features, and outcomes were evaluated.

**Results:**

Out of 294 patients, 18 patients had LVNC, with a prevalence of 6.1%. The mean age was 32 ± 13 years, and the majority were males (78%). The mean EF by echo was 36 ± 14 and by CMR was 31 ± 16 and the mean LV mass was 151 g. The mean LVEDV was 290 ± 154 and the mean LVESV was 211 ± 126. LGE was present in 33% of patients. The majority had uniform LV non-compaction (56%) followed by predominantly anterolateral and apical involvement (28%). Mitral regurgitation was the most common valvular pathology (33%). On follow-up of 37 months, the majority experienced at least one all-cause MACE (69%), while 14% of patients experienced mortality on follow-up. When compared with dilated cardiomyopathy patients without LVNC, the subjects were younger (p = 0.002) and had higher EF by an echocardiogram (0.001) and a lower arrhythmia hospitalization (p = 0.039). No difference was observed in overall MACE outcomes, mortality, and CMR features.

**Conclusion:**

The prevalence of LVNC is low in the studied population. Patients with LVNC have younger age, higher EF by echocardiogram, and lower arrhythmia hospitalization when compared with patients with dilated cardiomyopathy without evidence of LV non-compaction. The presence of LVNC does not confer an increased risk of MACE.

## Highlights

• Patients with LVNC have younger age, higher EF by echocardiogram, and lower arrhythmia hospitalization when compared with patients with dilated cardiomyopathy without evidence of LV non-compaction.
• The presence of LVNC does not confer an increased risk of MACE.

## 1 Introduction

Left Ventricle Non-Compaction Cardiomyopathy (LVNCC) is a type of myocardial disease characterized by prominent myocardial trabeculae and recesses resulting in two distinct layers of the myocardium: the compacted layer and the non-compacted layer. It arises due to the failure of left ventricle (LV) maturation and compaction during intrauterine life [1]. As per the position statement from the European Society of Cardiology, LVNCC has been labeled as an ‘unclassified’ type of cardiomyopathy [2]. The clinical course can be complicated by heart failure, thromboembolism, or arrhythmia [1].

**Fig. 1:**
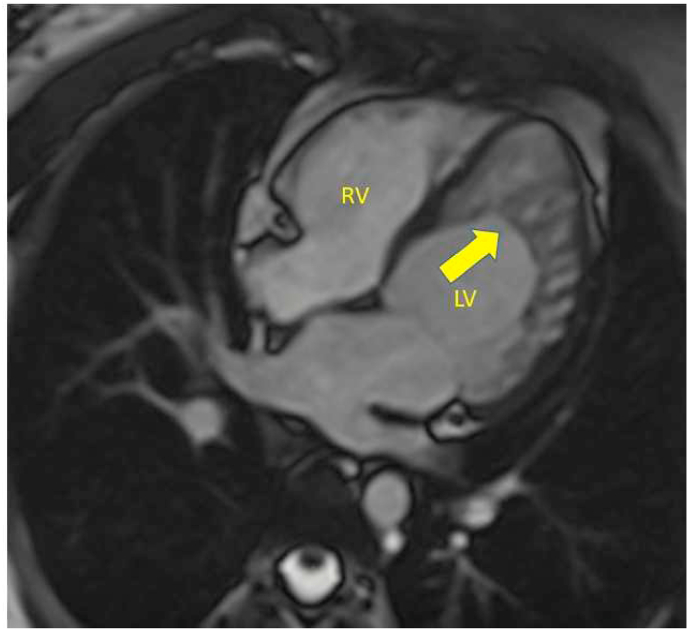
Cardiac magnetic resonance imaging steady-state free precession still-frame 4-chamber view showing left ventricle non-compaction (arrow). LV left ventricle, RV right ventricle.

Phenotypic presentation of LVNC can range from an extremely thickened layer of non-compacted myocardium to the mere presence of prominent trabeculae and recesses albeit a compacted myocardium [3]. Transthoracic echocardiography is the first tool to diagnose LVNC. However, Cardiac Magnetic Resonance (CMR) imaging has emerged as a strong tool to differentiate LVNCC from mere hyper-trabeculated LV myocardium when an echocardiogram is inconclusive (Fig. 1). Various criteria have evolved to diagnose LVNC by CMR. Peterson et al. defined the end-diastole non-compacted to compacted myocardium ratio of >2.3 to have good sensitivity, specificity, and negative predictive value for differentiating pathological non-compaction from hyper-trabeculation [4].

With an increase in the use of diagnostic cardiovascular imaging modalities in the South-Asian (SA) belt, LVNC is being increasingly diagnosed. The exact prevalence of LVNC in SA is not known and phenotypic CMR characteristics, clinical features, and outcomes of LVNC remain unknown for the SA population. This brought us to the need of analyzing the CMR data of LVNC at our center.

## 2 Methodology

We retrospectively enrolled 294 patients referred for CMR from 2011 to 2020 for evaluation of cardiomyopathy. Of these, 51% were ischemic cardiomyopathy, 26% were dilated cardiomyopathy, 25% were hypertrophic cardiomyopathy, 13% were restrictive cardiomyopathy, and 6% were arrhythmogenic right ventricle dysplasia. For final enrollment, patients with intracardiac masses, pericardial diseases, and congenital heart diseases were excluded from the study. Patients with evidence of LV non-compaction were further analyzed for baseline characteristics, CMR parameters, and outcomes. Characteristics of this group were compared with 47 patients who had dilated cardiomyopathy without evidence of LV non-compaction.

CMR was performed using a 1.5 T S Avanto Scanner with a breath-hold steady-state free precision sequence performed for every patient. Serial short and long-axis views were acquired using the following parameters: a slice thickness of 7 mm, a distance factor of 25%, a field of view of 34 cm, a matrix of 192 × 192, a flip angle of 80, a TR/TE of 58.74/1.12, and a bandwidth of 930 Hz/px. LGE images were acquired after 8–10 min of gadolinium injection. Third-party software was used to analyze all images (Media Q mass). Peterson criterion was used to diagnose LVNC.

Data analysis was done using Statistical Package for Social Sciences (SPSS) version 23.0.0 (IBM Corp. Released 2018). Results were presented as mean ± standard deviation for continuous variables such as age and LV volume and as a percentage for categorical variables. Baseline and CMR characteristics were recorded for all patients. Patients were followed up for any major adverse cardiovascular events (MACE) which included all-cause mortality, heart failure hospitalization, arrhythmia hospitalization, and cardiac implantable electronic device implantation on follow-up. An independent *t*-test was used for continuous variables and a chi-square test was used for qualitative data. A two-sided *P* < 0.05 was considered statistically significant for all tests.

Ethical approval was obtained from the ethical review committee of the hospital. The work has been reported in line with the STROCSS criteria [5]. This study has been registered with clinicaltrial.gov (UIN NCT05281315).

## 3 Results

Out of 294 patients referred for CMR for evaluation of cardiomyopathy, 18 patients were found to have LVNC, with a prevalence of 6.1%. The mean age was 32 ± 13 years, and males constituted most subjects (78%). The most common presenting complaint was dyspnea (95%), followed by palpitations (22%). Of note, none of the patients had a history of stroke. The mean EF by echocardiogram was 36% (Table 1).

**Table 1:** Baseline characteristics and echocardiographic features of patients with non-compaction on cardiac magnetic resonance imaging. SCD sudden cardiac death, EF ejection fraction, LVEDD left ventricle end-diastolic diameter, LVESD left ventricle end-systolic diameter, IVS interventricular septum.

Baseline characteristics	N = 18 (%)
Age (years)	32 ± 13 (Range 11–65)
Gender (Male)	14 (77.8)
Family history of cardiomyopathy	1 (6)
Family history of SCD	1 (6)
Dyspnea	17 (94.4)
Palpitation	4 (22)
Syncope/Presyncope	3 (19)
Diabetes Mellitus	0 (0)
Hypertension	1 (6)
History of stroke	0 (0)
Chronic Kidney Disease	0 (0)
Dyslipidemia	0 (0)
**Echocardiographic characteristics**	
EF	36 ± 14
LVEDD	50 ± 11
LVESD	39 ± 12
IVS thickness	9 ± 2
Posterior wall thickness	9 ± 2

**Fig. 2:**
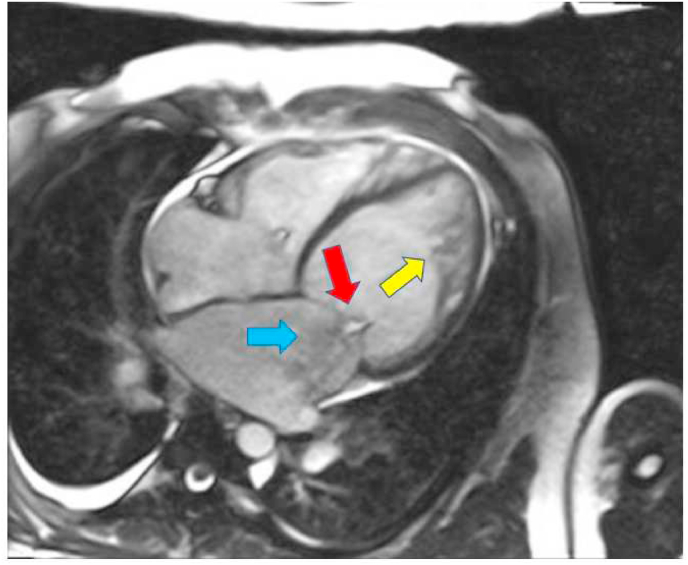
Cardiac magnetic resonance imaging steady-state free precession still-frame 4-chamber view showing left ventricle non-compaction (yellow arrow), mal-coaptation of the mitral valve (red arrow), and mitral regurgitation jet (blue arrow). Left ventricle volumes are increased. (For interpretation of the references to colour in this figure legend, the reader is referred to the Web version of this article.)

**Fig. 3:**
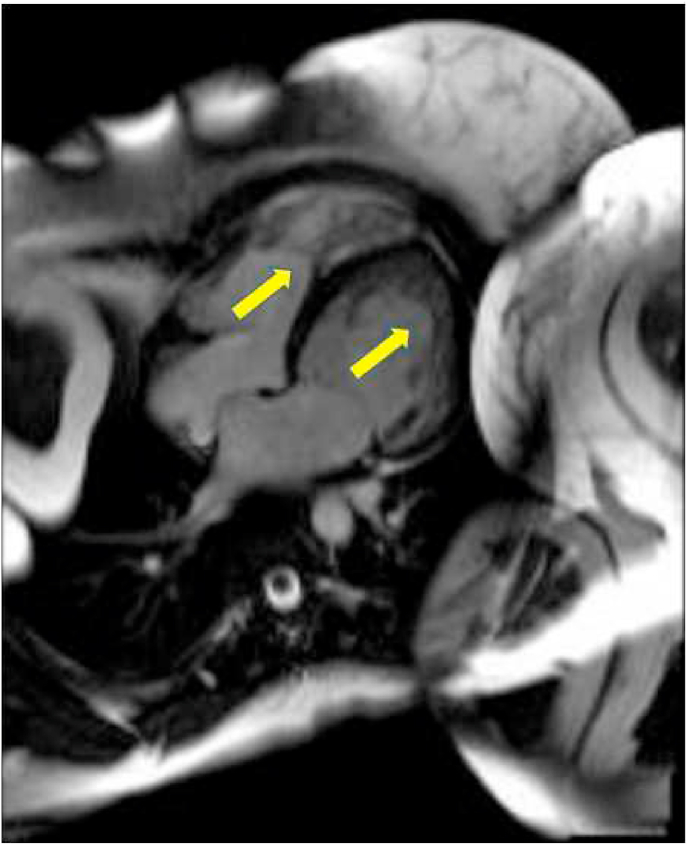
Cardiac magnetic resonance imaging steady-state free precision still-frame 4-chamber view showing left and right ventricle non-compaction (arrows).

The mean EF by CMR was 31% and the mean LV mass was 151 g. The majority (89%) had global hypokinesia. LGE was found in 33% of patients (Table 2). The distribution of non-compaction was as shown in Table 3. Mitral regurgitation (Table 3) was the most common valvular pathology associated with LVNC (33%) (Fig. 2). Right ventricle involvement was found in 2 patients (11%) (Fig. 3).

**Table 2:** Cardiac Magnetic Resonance characteristics of patients with evidence of non-compaction. EF ejection fraction, LVEDD left ventricle end-diastolic diameter, LVESD left ventricle end-systolic diameter.

CMR characteristics	N = 18
LVEDV	290 ± 154
LVESV	211 ± 126
Stroke volume	79 ± 52
EF (%)	31 ± 16
LV mass	151
RV enlarged	2 (11)
RV systolic dysfunction	3 (17)
Global hypokinesia	16 (89)
Pericardial effusion	2 (11)
Myocardial edema	1 (6)
Late gadolinium enhancement	6 (33)
Thrombus by CMR	1 (6)
Non-compaction criteria fulfilled	11 (61)
Non-compaction criteria not fulfilled	7 (39)

**Table 3:** Distribution of non-compaction on Cardiac Magnetic Resonance imaging and associated valvular pathologies. LV left ventricle, MR mitral regurgitations, AR aortic regurgitation. M male, F female.

Preferential distribution of non-compaction	N (%)
Both ventricles	2 (11)
LV (uniform distribution)	10 (56)
Antero-lateral and apical	5 (28)
Apical	1 (6)
**Valvular involvement**	**N (%)**
Mitral regurgitation	6 (33)
Aortic regurgitation	1 (6)

Follow-up was available for 16 patients (89%). On a mean follow-up of 37 ± 31 months, mortality was observed in 2 patients (14%). A total of 69% of subjects observed all-cause MACE on follow-up. The majority had at least one hospitalization for CV reasons on follow-up. The most common reason for hospitalization was heart failure (60%) followed by arrhythmia (32%) (Table 4).

**Table 4:** Outcomes of patients with LV non-compaction. CIED Cardiac implantable electronic devices, CV Cardiovascular, MACE major adverse cardiovascular events, LV left ventricle.

Outcomes on follow-up	
Mean duration of follow-up (Months)	37 months
All-cause mortality on follow-up (%) N = 14	2 (14)
MACE on follow-up (%) N = 16	11 (69)
CIED on follow-up	2 (11)
Mean CV hospitalization	1.56 ± 1.8
Total CV hospitalization	25
Total arrhythmia hospitalization (%)	8 (32)
Total heart failure hospitalization (%)	15 (60%)
Lost-to-follow-up	4 (22)

Patients with LV non-compaction were compared with patients with dilated cardiomyopathy (DCM) without evidence of LV non-compaction. Patients with LVNC were younger (p 0.002) and had higher EF by an echocardiogram (p 0.001). No statistically significant difference was observed for EF by CMR, presence of LGE, LV mass, and volumes. Patients without LVNC had lower arrhythmia hospitalization but no difference was observed in heart failure and total hospitalizations. All-cause MACE did not differ significantly between the two groups (Table 5).

**Table 5:** Comparison of patients with LV non-compaction with DCM patients with no evidence of LV non-compaction. EF ejection fraction, LVESV left ventricle end-systolic volume, LVEDV left ventricle end-diastolic volume, LV left ventricle, LGE late gadolinium enhancement.

Characteristics	Non-compaction of any degree	DCM with no evidence of non-compaction	P-value
Age (years)	29	41	0.002
EF by echocardiogram	44%	24%	0.001
EF by CMR	31%	28%	0.47
LVEDV by CMR	276	229	0.06
LVESV by CMR	209	168	0.09
LV SV by CMR	67	61	0.266
LV mass by CMR	144.8	144.7	0.996
Mean CV hospitalization	1.17	1.85	0.113
Mean heart failure hospitalization	0.78	0.79	0.976
Mean arrhythmia hospitalization	0.33	0.94	0.039
All-cause MACE	67	79	0.346
LGE	33	51	0.269

## 4 Discussion

LV non-compaction is a poorly understood entity. The exact prevalence and prognostic significance of this entity remain largely unknown. Recent years of CV imaging have witnessed an increase in the diagnostic rate of LVNCC due to the increased use of CMR. There is a lack of large, prospective, and conclusive data regarding the prognostic significance and the clinical presentation. This entity carries a tendency of over-diagnosis and higher false-positive rates associated with various diagnostic criteria and types of imaging modality used, as much as, it is not possible to state the exact prevalence of LVNC.

A large meta-analysis of 59 studies of LVNC was done by Ross et al. In this analysis, 26 cohorts were diagnosed using CMR, and Peterson criteria was the most used one. The prevalence of LVNC by CMR was 14.79% (95% CI 8.85–21.85) versus 1.28% (95% CI 0.95–1.64) by echocardiography [6]. However, this analysis did not include any South-Asian country. In our study from a South Asian tertiary care center, the overall prevalence of LV non-compaction in patients referred for CMR for evaluation of cardiomyopathy was 6.1%. According to the results of a study by Ivanov et al., whereby in 700 patients referred for CMR, LVNC had a prevalence of 39% by Petersen criteria (Also used in our study) [7]. This indicates some geographical and racial differences affecting the epidemiology of this entity. When compared with the LVNC cohort from North Carolina by Ivanov et al., our patients had a younger age of diagnosis and lower EF but comparable percentages of LGE and valvular involvement [7].

**Fig. 4:**
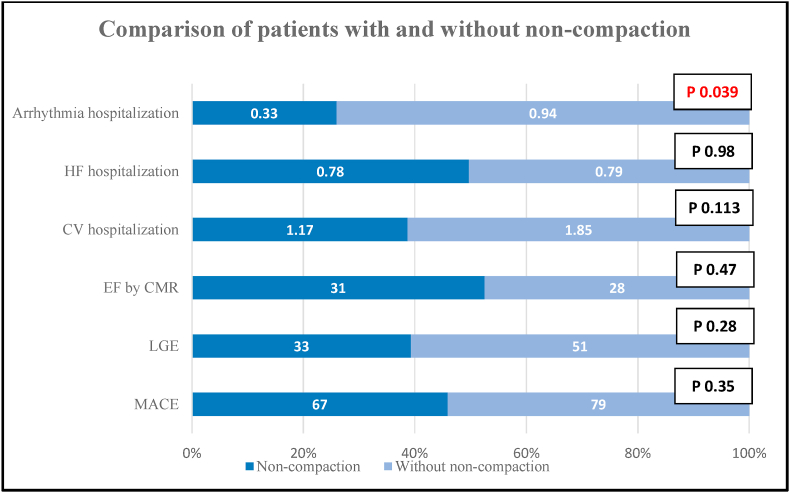
Comparison of patients with and without non-compaction. HF heart failure, CV cardiovascular, MACE major adverse cardiovascular event, LGE late gadolinium enhancement, EF ejection fraction.

We compared our group of LVNCs with DCM patients without evidence of non-compaction (Table 5 and Fig. 4). Our patients with non-compaction were strikingly younger (p = 0.002). Presence of non-compaction of any degree did not predict all-cause MACE (p = 0.346) or heart failure hospitalization (p = 0.976). However, patients without non-compaction had a significantly higher rate of arrhythmia hospitalizations (p = 0.039). Of note, EF by echocardiogram was higher in patients with non-compaction (p = 0.001). However, this difference was non-significant by CMR (p = 0.47). The possible cause of higher arrhythmia hospitalization in patients without non-compaction could be because of lower EF and a higher percentage of LGE in this group, although not reaching the level of statistical significance. Hence, it is very likely that predictors of outcomes in LVNC remain the same as for any cardiomyopathy such as LGE, LV EF, and LV stroke volume [8].

The association of mitral regurgitation (MR) and LVNC in the presence of normal EF is previously described in the literature [9,10]. Histologically, the pathologies include myxomatous degeneration, mitral valve (MV) leaflet sclerosis, and endocardial fibroelastosis. Phenotypic presentations include poor leaflet coaptation with zig-zag deformity, retracted leaflet, restricted movement of the leaflet, elongated chordae, and papillary muscle involvement. In our study (Table 3), MR was the most common valvular pathology associated with LVNC (6 out of 18, 33%). 3 out of 18 patients, had mal-coaptation of the mitral valve resulting in moderate-severe MR (Video 1), one patient had immobile posterior MV leaflet, and one patient had mitral valve prolapse (MVP). M Ali et al. prospectively studied 19 patients who had MR in the setting of LVNC with EF >45% by echocardiography. They found leaflet retraction in all patients and zig-zag deformity and mal-coaptation in 57% of patients and ruptured chordae in 15% of patients [10].

CMR provides an exact estimation of volumes and additionally defines alternative etiology. It also provides prognostic information. This study paves way for future CMR-based research in the country where economic-driven constraints restrict the use of CMR, even when indicated. Prospective studies are needed at a larger scale to estimate the overall outcomes of this disease entity in this part of the world.

## 5 Study limitations

This was a single-centered study. We were limited by the number of patients undergoing CMR due to cost constraints.

## 6 Conclusion

The prevalence of LVNC is low in the studied population. Patients with LVNC have younger age, higher EF by echocardiogram, and lower arrhythmia hospitalization when compared with patients with dilated cardiomyopathy without evidence of LV non-compaction. The presence of LVNC does not confer an increased risk of MACE. Overall, the predictors of all-cause in patients with LVNC likely remain to be the same as for any cardiomyopathy (such as LGE and EF).

## Sources of funding

No funding acquired for this study.

## Ethical approval

Ethical review committee of the Aga Khan University Hospital.

ERC number 2020-5594-14863.

## Consent

Consent not applicable as no direct intervention or interaction with human subjects.

## Author contribution

PS: Data management and analysis, literature search, and manuscript writing.

FAT: Supervised from conceptualization to final execution and manuscript writing.

## Registration of research studies

NOT APPLICABLE.

1. Name of the registry: clinicaltrial.gov
2. Unique Identifying number or registration ID: NCT05281315.
3. Hyperlink to your specific registration (must be publicly accessible and will be checked):

## Guarantor

Dr. Fateh Ali Tipoo.

## Provenance and peer review

Not commissioned, externally peer-reviewed.

## Declaration of competing interest

None of the authors has any conflict of interest to reveal.
