# Supplementary material for: Cardiac magnetic resonance features and outcomes of patients with non-compaction cardiomyopathy – A retrospective follow-up from Pakistan
Source: Ann Med Surg (Lond). 2022 Jun 17;80:103962. doi: 10.1016/j.amsu.2022.103962 (PMC9283803; doi:10.1016/j.amsu.2022.103962)
Supplement: Multimedia component 2 [file mmc2.docx]

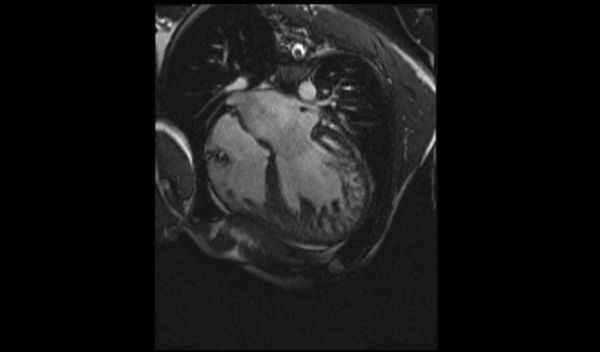


**Video 1**: Cardiac magnetic resonance steady-state precession free 4-chamber cine images showing left ventricle non-compaction predominantly in apical and anterolateral segments. Also, shown is mal-coaptation of the mitral valve and mitral regurgitation. The left atrium is dilated.
